# Cognitive abilities and medical students’ practice of physical exams: A quasi-experimental study

**DOI:** 10.1590/1516-3180.2022.0564.R1.10042023

**Published:** 2023-11-20

**Authors:** Lucas Moura Yamamoto, Matheus Landi Pavin, Giordano Bruno Duarte de Souza, Julio Lamartine Hayne Bastos de Oliveira, Raphael Raniere de Oliveira Costa, Adriano Yacubian Fernandes, Alessandra Mazzo

**Affiliations:** I Undergraduate Medical Student, Faculdade de Odontologia de Bauru (FOB), Universidade de São Paulo (USP), Bauru (SP), Brazil.; II Undergraduate Medical Student, Faculdade de Odontologia de Bauru (FOB), Universidade de São Paulo (USP), Bauru (SP), Brazil.; III Undergraduate Medical Student, Faculdade de Odontologia de Bauru (FOB), Universidade de São Paulo (USP), Bauru (SP), Brazil.; IV Undergraduate Medical Student, Faculdade de Odontologia de Bauru (FOB), Universidade de São Paulo (USP), Bauru (SP), Brazil.; V MD, MSc, PhD. Adjunct Professor, Medicine Course, Escola Multicampi de Ciências Médicas (EMCM), Universidade Federal do Rio Grande do Norte (UFRN), Caicó (RN), Brazil.; VI MD, PhD. Associated Professor, Faculdade de Odontologia de Bauru (FOB), Universidade de São Paulo (USP), Bauru (SP), Brazil. Associated Professor, Faculdade de Medicina de Botucatu, Universidade Estadual Paulista (UNESP), Bauru (SP), Brazil.; VII PhD. Associate Professor, Medicine Course, Faculdade de Odontologia de Bauru (FOB), Universidade de São Paulo (USP), Bauru (SP), Brazil.

**Keywords:** Physical examination, Students medical, Education, medical, Self-concept, Clinical skills laboratory, Students’ performance, Self-confidence, Simulation, Medical education, Simulation laboratory

## Abstract

**BACKGROUND:**

To highlight the importance of clinical simulations and simulated laboratories for student training, especially in physical examination teaching.

**OBJECTIVE:**

To evaluate the gains obtained by medical students in their cognitive and practical performance of physical examinations (abdominal, cardiological, and pulmonary), as well as satisfaction and self-confidence in what they have learned, after concentrated practice developed in a skills and simulation laboratory.

**DESIGN AND SETTING:**

A quantitative and quasi-experimental study in which 48 students were evaluated at the Faculdade de Odontologia de Bauru, São Paulo, Brazil.

**METHODS:**

A quantitative and descriptive study was conducted with regularly enrolled 2^nd^ year medical students over 18 years of age who had content prior to data collection regarding anamnesis and physical examination remotely taught in a Moodle virtual learning environment. For data collection, the participants were subjected to a concentrated period of skill training (abdominal, cardiological, and pulmonary). Every day after the skill training session, they were subjected to a practical evaluation and completed a theoretical test before and after the practical activities. At the end of all activities, they answered the instrument to assess the simulated practices (self-confidence and satisfaction).

**RESULTS:**

Among the 49 students evaluated, positive and significant theoretical and practical gains were identified in all three components (abdominal, cardiological, and pulmonary) (P = 0.000), as well as in the general evaluation (Theoretical 1 and Theoretical 2) (P = 0.000), satisfaction, and self-confidence (P = 0.000).

**CONCLUSION:**

Concentrated laboratory practice resulted in positive improvements in students’ physical examination skills.

## INTRODUCTION

There is a progressive resumption in the discussion of the relevance of studying semiology and semiotechniques within the medical routine, and the best way and time for students to learn this content is in undergraduate courses.

The etymology and definition of the terms “semiology” and “semiotechnique” are similar and related to the study of signs. They involve technical development and interpersonal relationships, which are consolidated through clinical experience. They analyze sign systems that communicate with humans and/or other beings from various perspectives. In medicine, semiology and semiotechnics provide subsidies for the professional to identify, rank, and interpret the semiological findings and confirm the existing symptoms through a general and specific physical examination, resorting to inspection, palpation, percussion, and auscultation techniques of the different body segments, in addition to additional tests. Its teaching requires the involvement of duly prepared professors capable of developing skills that are part of routine medical work for students.^1,2^

In the most diverse areas, skills and simulation laboratories have been cited as fruitful environments to learn these skills because, in an interactive way and with creative methods such as the use of simulators and/or other technologies, they stimulate the students’ experience in content that they consider difficult to understand.^3^

Physical examination has an incomparable potential in discovering problems, as it indicates diagnoses earlier in time without the need for supplementary clinical tests. In addition, verbal and non-verbal communication and also the contact between physicians and patients enhances their mutual trust and empathy.^4^

Learning the physical examination is complex, and in most medical courses in Brazil, it is taught almost entirely from the fifth semester of undergraduation,^5^ assuming that students possess enough knowledge to learn it only after this period. Scholars also report that the content has been taught in a remote learning style,^6^ which characterizes the passive mode of practical learning in which the professor demonstrates and the student observes, reproducing the sequence in a repetitive manner.

The medicine courses that resort to active methodologies are based on experimental knowledge to reconstruct knowledge.^7,8^ In this modality, students have contact with clinical practice and other teaching methods from the first semester, which assists them in better skill development.^7^ The use of clinical simulation stands out among the various methods that resort to experimental learning.

Clinical simulation mimics clinical practice in a safe environment. It can be performed with a series of resources, for example simulators and simulated patients.^4,8-11^ The difference between clinical simulation and a real practical situation is undeniable, and it is clear that reality is fundamental for training in medicine. However, several studies show that, when properly formulated and used, it can replace clinical practices by up to 50.0% without prejudice to the training quality.^11^ In addition, prior skills training increases patient safety^12^ and students’ self-confidence, security, and ease both in the scenarios and in real practice, which minimizes the patients’ unpleasant sensation of feeling like a learning object.

Skills and simulation laboratories are suitable environments for conducting physical examinations. However, some points still need to be clarified on this subject matter, such as the best resources to be used (practices with actors, role play, simulators, and other technologies) and how to distribute the activities within the curriculum of the courses (performing them sequentially, concentrated in a single block, one day after the other until they are finished, or throughout the semester and/or even the academic year, alternating between those with activities for inclusion in clinical practice).

## OBJECTIVE

This study aimed to evaluate the gains obtained by medical students in their cognitive and practical performance of the physical examination (abdominal, cardiological, and pulmonary) and to examine their satisfaction and self-confidence on acquiring this knowledge following concentrated practices developed in a skills and simulation laboratory.

## METHODS

### Type of study

This is a quasi-experimental study. Ethical approval was obtained from the Research Ethics Committee of Hospital de Reabilitação de Anomalias Craniofaciais (HRAC) on February 26, 2021 (Opinion no. 4.562.615). This study adhered to all ethical principles.

### Study locus

This study was conducted with students from a medical course at a public university in the inland of São Paulo, whose political-pedagogical project is based on an active methodology, has structured skills, and a simulation laboratory.

During the isolation period imposed by the coronavirus disease (COVID-19) pandemic in 2020, the teaching of semiology and semiotechniques underwent modifications in the course, which starts in the second semester. The theoretical contents of the anamnesis and the physical examination were taught exclusively through synchronous theoretical activities. Videos demonstrating the techniques were made available to the students in the study material of the virtual learning environment. When the students returned from the isolation period (beginning of 2021), strictly following adequate biosafety standards for their own protection,^13^ the practices were performed in groups and in a concentrated manner. This was also the students’ first contact with the unit, professors, facilitators, technicians, and peers of other courses.

### Population and sample

The participants were students attending second year of the course. The sample included those regularly enrolled, over 18 years old, entering 2020, who had anamnesis and physical examination content taught prior to data collection exclusively remotely, and who participated in all the study activities (theoretical test before the practical activities [Theoretical 1]; practical activities of abdominal, cardiac, and respiratory physical examination; practical evaluation of the abdominal, cardiac, and respiratory physical examination; and final theoretical test [Theoretical 2]). Students who did not perform any of the proposed activities were excluded. Therefore, 49 of 56 students in this group were included in the sample.

### Collection instruments

a) Instrument to characterize the subjects: An instrument with open and closed questions to characterize the students (course period, age, previous experiences with other undergraduate courses) and their behavior, as well as the resources used during the pandemic (access to the contents, interests, and availability of resources).
  Knowledge assessment (Theoretical 1 and Theoretical 2): an instrument created by the researchers themselves, subdivided into the contents of the cardiac, respiratory, and abdominal physical examination (Theoretical Abdomen, Theoretical Cardiology, Theoretical Respiratory). The instrument contained 10 multiple-choice questions for each content (cardiac, respiratory, and abdominal), with four answer options, of which only one was correct. The value assigned to each correct answer was 0.1. Before the application, the instrument was peer-validated regarding the face and content and tested a pilot stage with third-year students. No modifications were made to the instruments. The concept of cognitive knowledge used in this study arises from the theoretical perspective of the Miller pyramid. The “knowledge,” which is the pyramid’s base, refers to the evaluation of how the students integrate previous knowledge to the new information. In turn, practical performance corresponds to “show how to do it,” applied to the evaluation of skills.^14^
b) Evaluated skills with a checklist (abdominal practice, cardiology practice, and respiratory system practice). Based on the scripts used in the training, an instrument with 10 items and a value of 0.1 were assigned to each item created by the researchers, which contained a positive answer (*performed*) and a negative answer (*not performed*) as possibilities. Before the application, the instrument was peer-validated regarding the face and content and tested on a pilot stage with five third-year students. No modifications were made to the instruments.
c) Scale of Satisfaction and Self-Confidence in Learning (SSCL):^15^ a 13-item, five-point Likert-type instrument was used. It was divided into satisfaction (six items) and self-confidence in learning (seven items) dimensions, already validated and culturally adapted for Portuguese (Portuguese version: *Cronbach’s Alpha* = 0.84).

### Development of the study

When resuming in-person activities, the students were subjected to a concentrated skill training period for physical examinations. The training was performed for three consecutive days. The training sessions lasted for three hours and were conducted in groups of a maximum of 10 students. Before the practical activities were initiated, on day one, all students were invited to participate in the study; answer a theoretical evaluation that included content related to the abdominal, cardiac, and respiratory systems (Theoretical 1); and then participate in the practical activities.

The practical activities were divided by day into the following topics, all developed on three consecutive days: Day 1, Abdominal System; Day 2, Cardiac System; and Day 3, Respiratory System. They consisted of a demonstration for the entire group of students, followed by skill training and feedback. The demonstration was always conducted by the same facilitator, who had expertise in physical examinations. Skill training was conducted using trained and calibrated monitors under teacher supervision. Auscultation simulators and role-playing were used during the training sessions. All participants were guided by a script previously prepared and validated by the researchers.

At the end of each day of practical activity, the students were individually subjected to a practical evaluation in charge of the calibrated student monitor with the support of a checklist for skills assessment (abdominal practice, cardiology practice, and respiratory system practice). The practical evaluation followed the Objective Structured Clinical Examination model and included inspection, palpation, percussion, and auscultation techniques for all systems evaluated.

At the end of day 3 and the last practical evaluation, all students completed the knowledge evaluation (Theoretical 2) again. At the end of the evaluation, they completed the SSCL scale.^15^

### Analysis and presentation of the results

The data were tabulated and analyzed with the aid of the IBM SPSS Statistics 24 (IBM Corp., Armonk, New York, United States), a software that seeks analytical and data application. Descriptive statistics, comparison of means (Student’s *t*-test), reliability analysis (SSCL)^15^ and correlation analyses (Pearson’s test) were performed. The SSCL^15^ was analyzed as proposed by the original authors, independent of satisfaction and self-confidence evaluations. The web data were tabulated and analyzed in tables and a discursive report.

## RESULTS

Of the 48 students (100%), 28 (57.0%) were female and 21 (42.0%) were male. The younger student was 18 years old, the oldest was 29 years old, the average age was 21.7 years old, and the median age was 21 years. Among them, two (4.0%) had already attended another undergraduate course: one (2.0%) in Veterinary Medicine and one (2.0%) in Physical and Biomolecular Sciences.

All 48 (100.0%) students stated that they enjoyed accessing the Internet during the pandemic. Of these, nine (18.0%) reported not attending all the virtual classes. The reasons described were a lack of self-organization and readaptation to the teaching modality in five cases (10.0%) and the feeling of demotivation due to learning-at-a-distance in four cases (8.0%).

Regarding experiencing difficulties related to learning anamnesis and physical examinations through remote teaching, 48 (98.0%) reported experiencing them. The reported difficulties included a lack of practical activities (33.0%), difficulty understanding the semiotechnics (18.0%), difficulty discussing doubts remotely (18.0%), lack of correction of the skills trained at home (16.0%), lack of motivation (8.0%), and difficulty accessing the materials (4.0%).

With regard to the descriptive results of the theoretical and practical evaluations, an improvement was observed in the mean of the theoretical test after the simulated practices in the assessments of the cardiac (theoretical cardiology) and respiratory systems (theoretical respiratory), but there was no improvement in the mean of the theoretical evaluation of the abdominal system (theoretical abdomen) and in the general mean of the theoretical evaluation (Theoretical 2); however, the standard deviation for the scores of all systems and of the general evaluation was reduced. An improvement in the mean grade corresponding to practical evaluation 2 (cardiology) was also observed compared with the mean grade for practical evaluation 1 (abdomen). The mean values across practical evaluations also showed a reduction in the standard deviation.

Considering the normal distribution of the sample (Kolmogorov-Smirnov ≤ 0.05), a means comparison was also made (Student’s *t*-test) between the subsequent theoretical grades and between the means of the practical evaluation and those of the final theoretical evaluation; a significant gain was observed among all the evaluations performed (**Table 1**).

**Table 1.** Gains obtained by the students between the theoretical and practical evaluations. Bauru, 2022

Evaluation	Fr	Min	Max	Mean	SD	*t-test*	*DoF*	P value
***Theoretical***								
Theoretical 1 Abdomen	49	0.3	1.9	1.3	0.375	24.018	48	0.000
Theoretical 2 Abdomen	49	0.6	1.6	1.1	0.254	49.233	48	0.000
Theoretical 1 Cardiology	49	0.3	1.7	1.0	0.322	22.361	48	0.000
Theoretical 2 Cardiology	49	0.7	1.9	1.4	0.271	36.660	48	0.000
Theoretical 1 Respiratory	49	0.0	2.0	1.1	0.412	19.223	48	0.000
Theoretical 2 Respiratory	49	1.0	2.0	2.0	0.172	79.781	48	0.000
Theoretical 1	49	0.6	1.6	1.1	0.254	31.495	48	0.000
Theoretical 2	49	0.5	1.3	1.0	0.153	45.972	48	0.000
***Practical***								
Practical 1 Abdomen	49	0.5	1.0	0.8	0.137	38.293	48	0.000
Practical 2 Cardiology	49	0.5	1.0	0.9	0.123	48.561	48	0.000
Practical 3 Respiratory	49	0.7	1.0	0.9	0.090	70.320	48	0.000

Fr = frequency; Min = minimum; Max = maximum; SD = standard deviation; DoF = degrees of freedom.

The SSCL was applied to assess the students’ satisfaction and self-confidence levels obtained in the simulated clinical practices during the period.^15^ The scale presented good data reliability (α = 0.833), and the comparison (Student’s *t*-test) between the means obtained showed that there were significant gains when comparing satisfaction and self-confidence (**Table 2**).

**Table 2.** Gains obtained by the students between the theoretical and practical evaluations. Bauru, 2022

SSCL	Fr	Min	Max	Mean	SD	*t*-test	*DoF*	P value
Satisfaction	49	3.3	5.0	4.3	0.518	57.706	48	0.000
Self-confidence	49	3.2	5.0	4.7	0.416	78.882	48	0.000

Pearson’s test was performed to verify whether there was any correlation between satisfaction and the evaluation results. The correlations found between the practical evaluation and scale (P = 0.218) and between the theoretical evaluation and scale (P = 0.196) were very weak.

## DISCUSSION

This study was conducted with students returning from the pandemic distancing period (COVID-19), and the results showed that although they had internet access during the suspension of their academic activities, they had difficulties related to the participation and development of practical activities. In the medical field, clinical experience with patients and physical contact between students are part of the development of competent professionals and are stimulated by the Curricular Guidelines of Undergraduate Courses. However, universities have been forced to review their teaching strategies during the pandemic. Many strategies that until then were exclusively based on the face-to-face issues started to introduce online content,^16^ including those related to videoconferencing, transforming face-to-face classes into synchronous ones, and others used e-learning strategies.^17^ During this period, some more interactive e-learning activities were the best evaluated and accepted by the undergraduate students.^18^

With regard to the semiology and semiotechnics practices supported by e-learning, initiatives that individualize teaching and learning methods by providing feedback to the students on their limitations and possibilities through contact with real clinical cases are already in progress, have been well evaluated, and show room for improvement.^2^ In the sample of this study, the physical examination was reported as the item that presented with the highest difficulty to be performed in the home environment, which can be related to the use of resources within a skill and simulation laboratory and to the constant support of a facilitator when such activities are performed in person. Supported by skills and simulation resources, physical examination has been an effective practice in both medical and other healthcare professions.^19^ Some researchers emphasize the importance of clinical practice at the bedside and recommend that to balance the difficulties experienced in the post-pandemic period, activities that revisit physical examination techniques in an arduous way and individually and/or in groups should be performed with clear and precise strategies to meet the needs.^20^

In this study, to meet the requirements of the physical examination training, several sessions of the abdominal, cardiological, and respiratory examinations were performed on subsequent days. As shown in **Table 1**, the results indicate a gain in knowledge among the students after all the activities and a significant increase in practical and theoretical knowledge in the subsequent days. The practices thus exerted a positive impact both on skill training and on the students’ cognitive performance.

Other studies have reported similar results regarding improvements in cognitive performance. Some time passed between practice and cognitive learning (theoretical classes were online during the COVID-19 pandemic in 2020); in this study, the association between students’ theoretical and practical knowledge and experimental learning^7^ demonstrated success. Although these findings need to be further explored in studies that control for other variabilities, such as online access to activities and study time, the results showed that reviewing theoretical content (cognitive learning^7^) should not be the exclusive strategy used by medical students. Students who learn by simulation learn more, and this knowledge lasts longer.^21,22^ By enabling students’ active participation, simulation presents itself as a strategy of greater impact for students when compared to more traditional strategies.^23^

In relation to practical performance, these findings are grounded in Kolb’s theories of Experiential Learning and on David Ausubel’s Theory of Meaningful Learning.^24^ Experiential learning is considered a continuous process based on reflection that begins when an individual is involved in a situation. This situation can be created as in the case of clinical simulation sessions. Experience enables subjects to change their behavior or attitude; has numerous benefits for education; and promotes emotional, behavioral, and cognitive learning.^9,10,24^ In addition, active experimentation can contribute to the development of competencies and skills.^24^

From the perspective of meaningful learning, knowledge is attributed to meaning when there is an interaction with previous knowledge. When interacting with new knowledge, previous knowledge modifies and enriches the previous cognitive structure. In simulations, prior knowledge is facilitated in earlier stages, such as concepts, data, information, theories, and skill practices. During a simulation session, there is a necessary interaction with previous knowledge, resulting in the attribution of meaning to knowledge and meaningful learning. Meaningful learning lasts longer.^26^

In terms of physical examination skills, there are difficulties and deficiencies in the qualifications and skills intrinsic to professionals, which makes individual training at both the undergraduate and graduate levels extremely relevant. In the cardiac physical examination, using simulators and skill and simulation laboratories have been recommended for medical education, and some studies have proven the effectiveness of skills and knowledge gained with these resources.^27^ Others even report that this can be a transforming element for patient care and also for medical education.^28^ In cardiology, some researchers suggest, for example, resorting to auscultation stations using deliberate practice to maximize the potential of such practice.^29^

Regarding pulmonary physical examination, this study shows good use of skill and simulation laboratories with simulators, in the sense of developing technical attributes, but also of interaction with the patient, respecting ethical and safety issues. Some researchers have moved in this direction by producing technical manuals on how to conduct such learning.^30^ In turn, in abdominal physical examination, the clinical skills of inspection, palpation, percussion, and auscultation, sometimes replaced in large centers by imaging examinations, are highlighted as extremely relevant in the support of bedside diagnoses, since the beginning of the medical studies.^31^

By applying the SSCL scale^15^ (**Table 2**), it was possible to identify gains in student satisfaction and self-confidence with the simulated practices. Satisfaction can be considered as a feeling of pleasure or disappointment that arises from an event and from the individual’s previous perspectives on satisfaction.^32^ It is an affective reaction caused by the occurrence of what is expected ahead.^33^ In simulated teaching, satisfaction can be considered an important component, mainly due to the positive reinforcement in self-confidence and in the experiences that will build the profile of future professionals.^21^ Satisfied students are more motivated to learn.^21,34^

Self-confidence is related to the beliefs demonstrated in the event, domains, and dexterities, and is performed with wisdom, preparation, and support.^35^ In simulation, satisfaction and self-confidence seem to be related to interactivity of the resources, refinement of skills, support from facilitators, and competence.^9^ A number of studies have pointed to simulation as a strategy that promotes satisfaction and self-confidence in students in the medical field.^36-40^

### Limitations of the study

The study had the following limitations: non-comparison between the gains obtained after concentrated practice and other practice models, such as interleaved practices, and those distributed throughout the semester. Therefore, it is not possible to infer which model is the most suitable for teaching anamnesis and physical examinations in relation to the segments studied.

Only immediate cognitive and practical knowledge, and performance were assessed. Cognitive performance, satisfaction, and self-confidence after the online classes were not measured. Therefore, it is necessary to develop prospective and experimental studies with the objective of longitudinally monitoring performance and assimilation using different models to organize and offer skill practices in medical education.

## CONCLUSION

In the cognitive performance, it was observed that the students presented positive and significant gains in all three components (abdominal, cardiological, and pulmonary) and in the general evaluation (Theoretical 1 and Theoretical 2). Regarding the practical evaluation, there was also a positive and significant gain among all three components.

Concentrated practice provided positive and significant gains in terms of satisfaction and self-confidence among medical students. However, it was not possible to identify a strong correlation between the cognitive performance and practical evaluations.

## COGNITIVE ABILITIES AND MEDICAL STUDENTS’ PRACTICE OF PHYSICAL EXAMS: A QUASI-EXPERIMENTAL STUDY

The São Paulo Medical Journal thanks Rodrigo Guimarães and Fernanda Miranda for their contributions to the peer review of this manuscript.

**Table:** PEER REVIEW REPORTS

Reviewer 1: Rodrigo Guimarães - Universidade Federal de Mato Grosso do Sul, Instituto Integrado de Saúde	
First evaluation	Second evaluation
Recommendation: Minor Revision Comments: The manuscript is interesting in terms of the teaching-learning process of physical examination, but it needs some adjustments and clarifications. The title is too long and unattractive, I suggest its reduction. A suggestion would be: COGNITIVE AND PRACTICAL SKILLS OF MEDICINE STUDENTS IN PERFORMING PHYSICAL EXAMINATION: A QUASI-EXPERIMENTAL STUDY In the abstract I suggest adding between the descriptors simulation and teaching to increase the probability of reaching the article. The introduction is coherent, as well as the applied study methodology. In the results, the authors bring income as a variable of interest, but it is very loose in the manuscript. Would the idea be to associate it with internet access? I suggest establishing consistency with this variable or removing it. In Tables 2 and 3 the values of P value are all zeroed, is there the result found? I suggest reviewing. The scale used and shown in Table 3 is composed of 13 items totaling 130 points with facts of 60 and 70 points. I suggest that the authors present, in addition to the frequencies of minimum, maximum and standard deviation, such results as well as the score obtained in the general score. The manuscript presents the limitations of the study and the conclusion is adequate. Additional Questions: Does the manuscript contain new and significant information to justify publication? Yes Does the Abstract (Summary) clearly and accurately describe the content of the article? Yes Is the problem significant and concisely stated? Yes Are the methods described comprehensively? Yes Are the interpretations and conclusions justified by the results? Yes Is adequate reference made to other work in the field? Yes Is the language acceptable? Yes Please rate the priority for publishing this article (1 is the highest priority, 10 is the lowest priority): 5. Length of article is: Adequate. Number of tables is: Adequate. Number of figures is: Adequate. Please state any conflict(s) of interest that you have in relation to the review of this paper (state “none” if this is not applicable): None Rating: Interest: 2. Good. Quality: 2. Good. Originality: 2. Good. Overall: 2. Good.	Recommendation: Accept The authors accepted the proposed improvements suggestions to the manuscript. Therefore, I suggest publishing. Additional Questions: Does the manuscript contain new and significant information to justify publication? Yes Does the Abstract (Summary) clearly and accurately describe the content of the article? Yes Is the problem significant and concisely stated? Yes Are the methods described comprehensively? Yes Are the interpretations and conclusions justified by the results? Yes Is adequate reference made to other work in the field? Yes Is the language acceptable? Yes Please rate the priority for publishing this article (1 is the highest priority, 10 is the lowest priority): 6. Length of article is: Adequate. Number of tables is: Adequate. Number of figures is: Adequate. Please state any conflict(s) of interest that you have in relation to the review of this paper (state “none” if this is not applicable): None Rating: Interest: 2. Good. Quality: 2. Good. Originality: 2. Good. Overall: 2. Good.
**Reviewer 2:**	
**Anonymous**	
**Did not authorize the publication of the peer review reports**	
**First evaluation**	**Second evaluation**
Recommendation: Major Revision	Recommendation: Accept
